# Effect of dose splitting of a low-volume bowel preparation macrogol-based solution on CT colonography tagging quality

**DOI:** 10.1007/s11547-022-01514-4

**Published:** 2022-06-17

**Authors:** Francesco Mistretta, Nicolò Damiani, Delia Campanella, Simone Mazzetti, Alessia Gulino, Giovanni Cappello, Daniele Regge

**Affiliations:** 1 Department of Surgical Sciences, University of Turin, Turin, Italy; 2 Radiology Unit, Candiolo Cancer Institute, FPO-IRCCS, Candiolo, Italy

**Keywords:** CT colonography, Fecal tagging, Bowel preparation, Quality assessment, Patient tolerance

## Abstract

**Purpose:**

To compare examination quality and acceptability of three different low-volume bowel preparation regimens differing in scheduling of the oral administration of a Macrogol-based solution, in patients undergoing computed tomographic colonography (CTC). The secondary aim was to compare CTC quality according to anatomical and patient variables (dolichocolon, colonic diverticulosis, functional and secondary constipation).

**Methods:**

One-hundred-eighty patients were randomized into one of three regimens where PEG was administered, respectively: in a single dose the day prior to (A), or in a fractionated dose 2 (B) and 3 days (C) before the examination. Two experienced radiologists evaluated fecal tagging (FT) density and homogeneity both qualitatively and quantitatively by assessing mean segment density (MSD) and relative standard deviation (RSD). Tolerance to the regimens and patient variables were also recorded.

**Results:**

Compared to B and C, regimen A showed a lower percentage of segments with inadequate FT and a significantly higher median FT density and/or homogeneity scores as well as significantly higher MSD values in some colonic segments. No statistically significant differences were found in tolerance of the preparations. A higher number of inadequate segments were observed in patients with dolichocolon (*p* < 0.01) and secondary constipation (*p* < 0.01). Interobserver agreement was high for the assessment of both FT density (*k* = 0.887) and homogeneity (*k* = 0.852).

**Conclusion:**

The best examination quality was obtained when PEG was administered the day before CTC in a single session. The presence of dolichocolon and secondary constipation represent a risk factor for the presence of inadequately tagged colonic segments.

## Introduction

Computed tomographic colonography (CTC) is an accurate and well-accepted, non-invasive method for colonic imaging [1]. One of the few downsides of CTC is bowel preparation which is often described by patients as the most uncomfortable part of the examination [2–7]. To improve tolerance, low-volume bowel preparations have been investigated [8–13] which however might compromise examination quality.

Polyethylene glycol (PEG) is a hydrophilic and iso-osmolar laxative that has few side effects since it is non-absorbable and does not alter the patient’s electrolyte balance. PEG has been proposed in low-volume solutions either in a single or fractionated dose [10–17]. A variable amount of residual fluid is usually present in the large bowel following PEG administration that can easily be tagged by oral intake of iodinated contrast media, which has been extensively evaluated [13, 18–20]. However, to our knowledge, previous studies have not investigated administration schedules for PEG intake. In particular, it is not known to date whether a single administration on the day before the examination provides better quality and acceptability than a fractionated dose spread out in 2 or 3 days.

Besides FT and PEG regimens, several other factors might affect examination quality, in particular patient-related variables such as colon length, the presence of diverticulosis or functional and secondary constipation.

The aim of this study was to compare examination quality and acceptability of three preparation regimens differing only for the administration schedule of a low-volume PEG-based solution, in patients undergoing CTC with fecal tagging. The secondary aim was to compare how CTC quality was affected by patient variables.

## Materials and methods

The local ethical committee authorized the study which was conducted in accordance with the declaration of Helsinki and national legislation.

### Study design and population

**Fig. 1:**
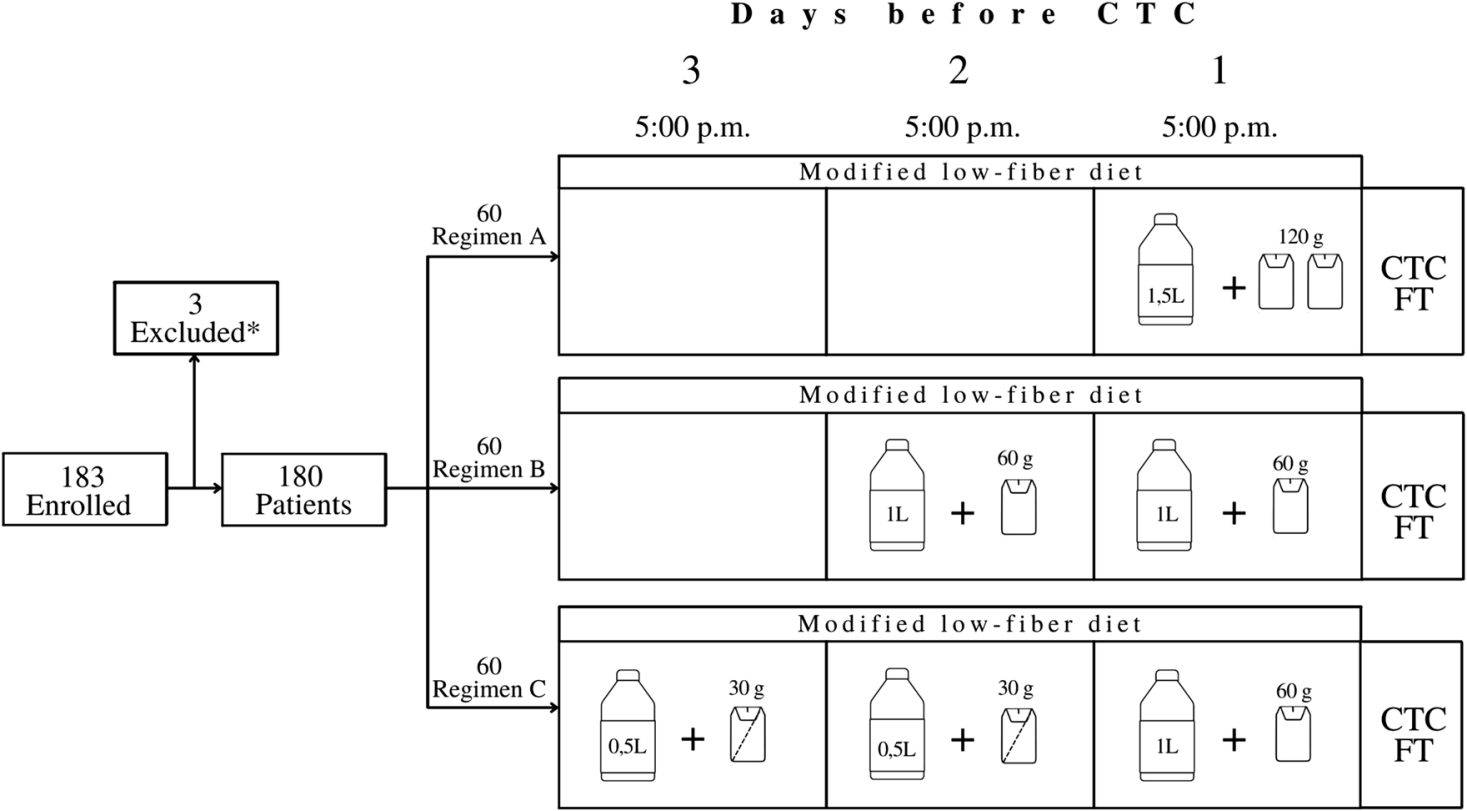
Study design and bowel preparation schemes. Regimen A: 120 g of PEG in 1.5 L of water at 5 pm on the day before the CTC; Regimen B: 60 g PEG in 1 L of water at 5 pm on the 2 days before the CTC; Regimen C: 30 g PEG in 0.5 L of water at 5 pm on the 3rd day before the CTC + 30 g PEG in 0.5 L of water at 5 pm on the 2nd day before the CTC + 60 g PEG in 1 L of water at 5 pm on the day before the CTC. CTC computed tomographic colonography, FT fecal tagging. **a**. Patients with indications to perform CTC in according to ESGE/ESGAR guideline consensus statement. **b**. Three patients were excluded because of indication to perform CTC with iodinated contrast media intravenously after prone scan, failure to follow the assigned preparation scheme and appearance of vasovagal reaction during CO_2_ insufflation

This single-center randomized prospective study enrolled 180 patients between September 2018 and April 2020. Inclusion criteria were indications to CTC according to the ESGE/ESGAR guideline consensus statement [1]. Exclusion criteria were bowel preparation scheme not respected, CTC performed with intravenous contrast medium and/or interruption of CTC examination (i.e., appearance of vasovagal reaction during CO_2_ insufflation). Patients were assigned to one of three different reduced bowel preparation regimens using a computer-generated random number to obtain 60 patients for each regimen (Fig. 1). Demographics data and patient characteristics are reported in Table 1.

**Table 1:** Demographics data and patients features

Regimen	Mean age, years (range)	Male/female Ratio	Dolichocolon, *n* (%)	Functional constipation, *n* (%)	Causes of secondary constipation, *n* (%)	Colonic diverticulosis, *n* (%)
A	67.5 (31–87)	18:42	26 (43.3)	6 (10)	16 (26.7)	21 (35)
B	67.6 (40–89)	29:31	21 (35)	13 (21.7)	3 (5)	29 (48.3)
C	66.5 (41–87)	20:40	27 (45)	13 (21.7)	8 (13.3)	22 (36.7)

### Bowel regimens and CTC image protocol

All patients were asked to comply with a modified low-fiber diet starting 3 days before the CTC. Bowel cleansing was performed using a low-volume iso-osmolar and isotonic PEG solution [Colonpeg®, Sanitas farmaceutici, Macrogol 3350 (99.98%), Sucralosio (< 0.1%)]. This tasteless and water-soluble solution was administered according to one of three preparation schemes shown in Fig. 1. On the day of examination, approximately 2.5 h prior to entering the CT room, all patients were given 70 ml of sodium diatrizoate and meglumine diatrizoate (Gastrografin®, Bayer Schering Pharma) diluted in 0.5 L water followed 30 min later by the administration of additional 0.5 L of still water.

CTC was performed after the introduction of a Foley catheter (20 Fr) in the rectum and pneumocolon was induced by insufflating CO_2_ with the use of an automatic device (PROTOCO2L TOUCH®; Bracco Imaging), until adequate distension was obtained or maximum patient tolerance reached.

All CT acquisitions were conducted on a 128-slice image CT system (SOMATOM DEFINITION FLASH; Siemens Healthineers) with scans in both the supine and prone decubitus positions (rotation time 0.5 s; collimation 128 × 0.6 mm; pitch 1; tube voltage 120 kV; tube current 30 mAs; section thickness 1 mm; reconstruction interval 0.7 mm; safire S3; kernel I26f). If colon distension was deemed insufficient, an additional lateral decubitus position was performed.

### Analysis of preparation quality

#### Qualitative assessment

**Fig. 2:**
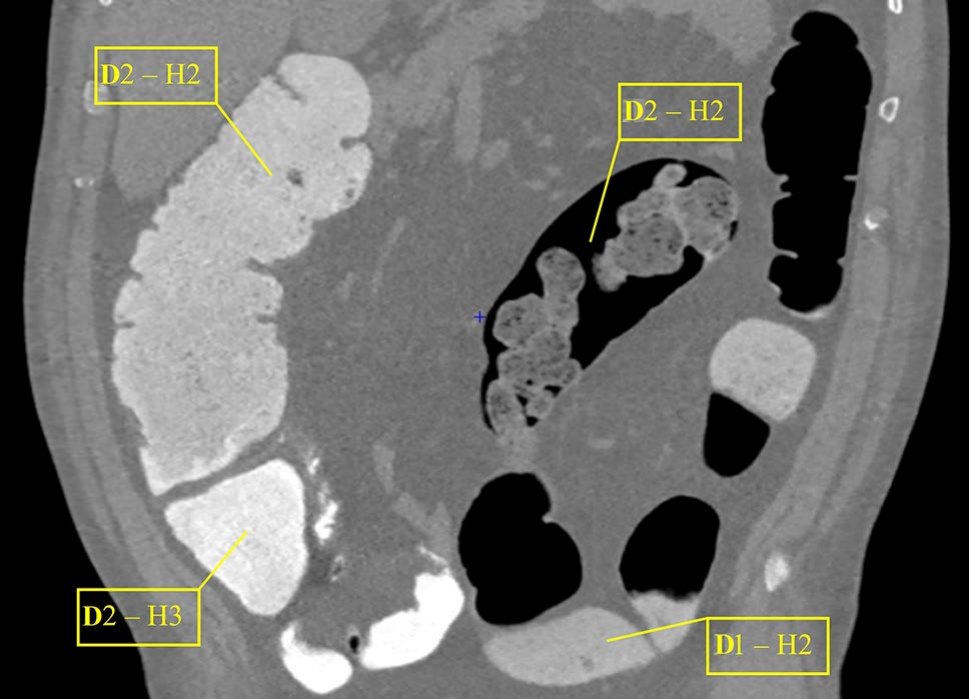
Coronal abdominal view of a poor-quality CTC in a patient with dolichocolon. The radiologists assigned a score of 2 for density and 3 for homogeneity of the FT in the caecum; the FT of other colonic segments was considered inadequate with decreasing score from ascending colon to sigmoid-rectum. *I* intensity score, *H* homogeneity score

Two experienced radiologists (> 500 CTC per year), blinded to the patient’s regimen, assessed the degree of homogeneity and tagging density. Analysis was performed on a per-segment basis, using the two scales shown in Table 2. For the purpose of the analysis, the large bowel was divided into the following six segments: caecum, ascending, transverse, descending and sigmoid colon, and rectum. Each radiologist assigned an overall per-segment score considering FT homogeneity and density. For each segment, the CT scan in the decubitus position with the best quality was considered for evaluation. When radiologists assigned different scores, a face-to-face review was performed for consensus. Colonic segment assessment was considered inadequately tagged if a score of 1 for density and/or of 1–2 for homogeneity was assigned by readers. In the per-patient analysis, CTC was considered “non-diagnostic” if at least one colonic segment was inadequately tagged (Fig. 2). The interobserver agreement was assessed for both FT density and homogeneity scores.

**Table 2:** Scales of subjective scores assigned by the two radiologists for density (a) and homogeneity (b) of fecal tagging (FT)

a		
Density score	Definition	FT
1	Distinction between colonic wall and FT is not possible	Inadequate
2	Contrast between colonic wall and FT is sufficient for a diagnostic assessment	Adequate
3	Optimal distinction between colonic wall and FT	

b		
Homogeneity score	Definition	FT
1	Solid or liquid fecal residues non-tagged	Inadequate
2	Partially tagged solid or liquid fecal residues that simulate polyps or do not allow evaluation of the colonic wall	
3	Partially tagged solid or liquid fecal residues that do not simulate polyps and allow evaluation of the colonic wall	Adequate
4	Optimal homogeneous tagging

#### Quantitative assessment

**Fig. 3:**
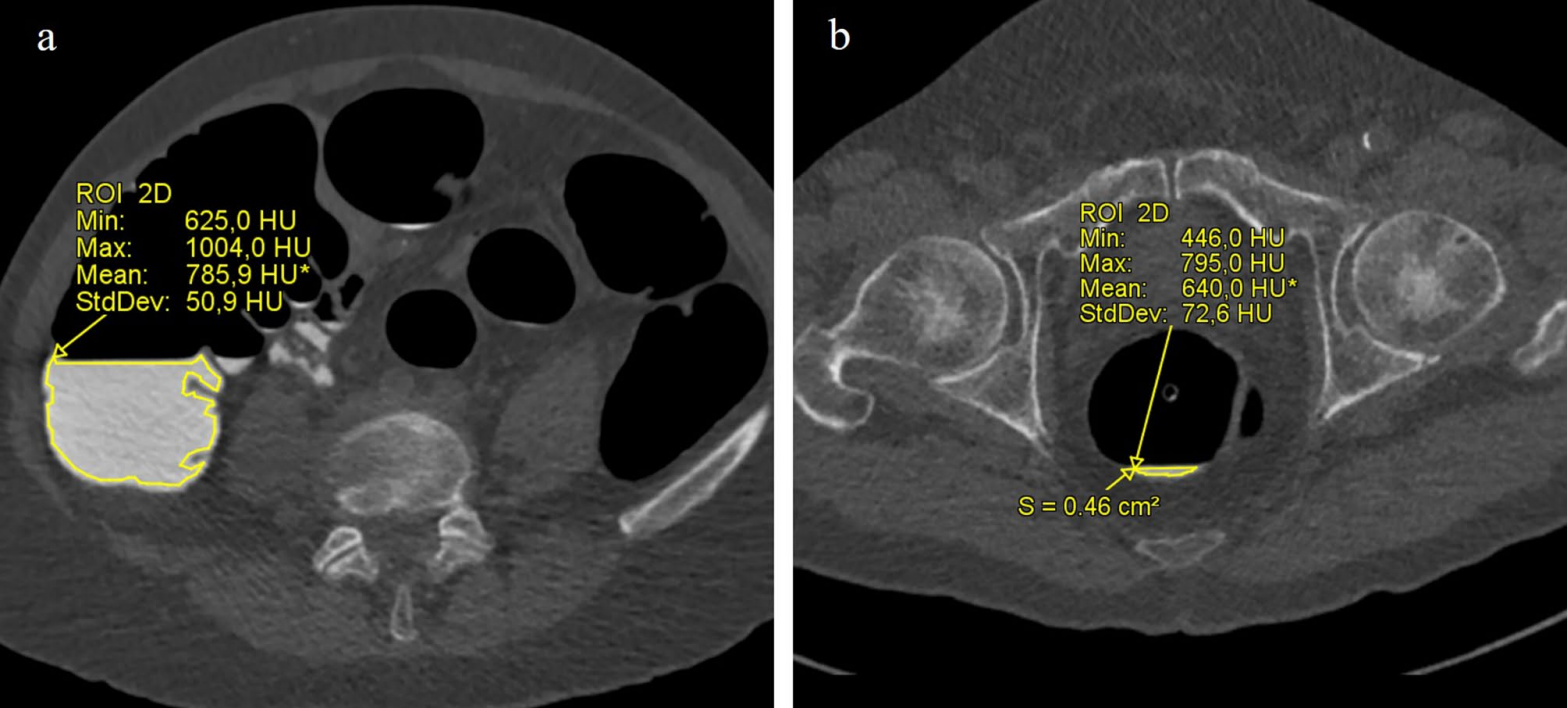
**a** Example of a manually drawn ROI to evaluate MSD and RSD of FT: an area that includes all fecal residues, excluding anatomical structures such as colonic mucosal folds. **b** The amount of fecal residues in the rectum was insufficient for the quantitative analysis (manually drawn ROI < 0.5 cm.^2^)

Mean segment density (MSD), measured in Hounsfield units, and relative standard deviation (RSD) were measured for the evaluation of the FT quality in the quantitative analysis. The first measure reflected the density of the FT and the second its homogeneity. Both values were obtained in each of the six colonic segments using a region of interest (ROI) manually drawn by one of the two radiologists on the decubitus where the greatest amount of tagged fluid was identified, excluding anatomical structures such as colonic wall and mucosal folds (Fig. 3a). Segments with fecal and/or liquid residues in an area less than 0.5 cm^2^ were excluded from the analysis since measurements were considered unreliable (Fig. 3b). To assess interobserver variability, the second radiologist, blinded to patient’s regimen and previously drawn ROIs by the other operator, manually drew a second set of ROIs in each of the six colonic segments in a subgroup of patients (the first ten subjects for each regimen).

### Patient acceptance

Each patient was asked to express the tolerance to the administered preparation regimen using a visual analogical scale, where a value of 0 corresponded to no discomfort and 10 to an extreme discomfort, with a negative impact on the patients’ daily activities.

### Impact of patient features on CTC quality

For each patient, the presence of colonic diverticulosis and dolichocolon was defined subjectively with visual evaluation in consensus by the radiologists. The presence of functional constipation (defined in according to Rome III Criteria) [21] or secondary constipation was recorded using a specific questionnaire.

### Statistical analysis

Subjects were randomized after signing informed consent into one of three regimens, following a block randomization with no stratification factors. The randomization list was computer-generated by an external randomization manager and investigators were blinded to regimen allocation.

Qualitative parameters in the per-segment and per-patient analysis were compared using the chi-squared test. At first, the three regimens were compared to identify the presence of statistically significant differences, then multiple comparisons across pairs were performed to identify the source of differences. On the per-segment basis, the null hypothesis was that all regimens had the same frequency of inadequate segments, while on a per-patient analysis, the null hypothesis was that all regimens had the same frequency of non-diagnostic CTC, against the alternative hypothesis that there was a significant difference between the frequencies of the three regimens.

Medians of the qualitative parameters were assessed for each regimen and segment. Comparisons were performed using the Kruskal–Wallis test and when a statistically significant result was found, multiple comparisons across pairs were performed using the Mann–Whitney test.

Quantitative parameters (MSD and RSD) were compared among the three different regimens using the Kruskal–Wallis test to verify the tagging quality throughout the colon. The null hypothesis was identical median for all regimens, against the hypothesis that the medians were not equal.

Interobserver variability was assessed between the two readers on both qualitative and quantitative parameters. FT density and homogeneity qualitative scores were compared using the Cohen’s kappa inter-rater agreement, with *k* = 1 meaning perfect agreement between the two readers and *k* = 0 when no agreement was found. 95% confidence intervals (95% CI) for the inter-rater agreement were also provided. Quantitative parameters were compared between the two readers using the Wilcoxon paired sample test, and the analysis was performed on both MSD and RSD measurements.

Patient acceptance to preparations were compared using the Kruskal–Wallis test to assess if one or more regimens were more tolerated than others.

Patient-dependent parameters (dolichocolon, constipation, cause of constipation and colonic diverticulosis) were analyzed for each single factor using the chi-squared test to evaluate differences between the adequate and inadequate segments.

A *p* value lower than 0.05 was used to define statistically significant results, and analyses were performed using MedCalc v18.6.

## Results

### Qualitative assessment

**Fig. 4:**
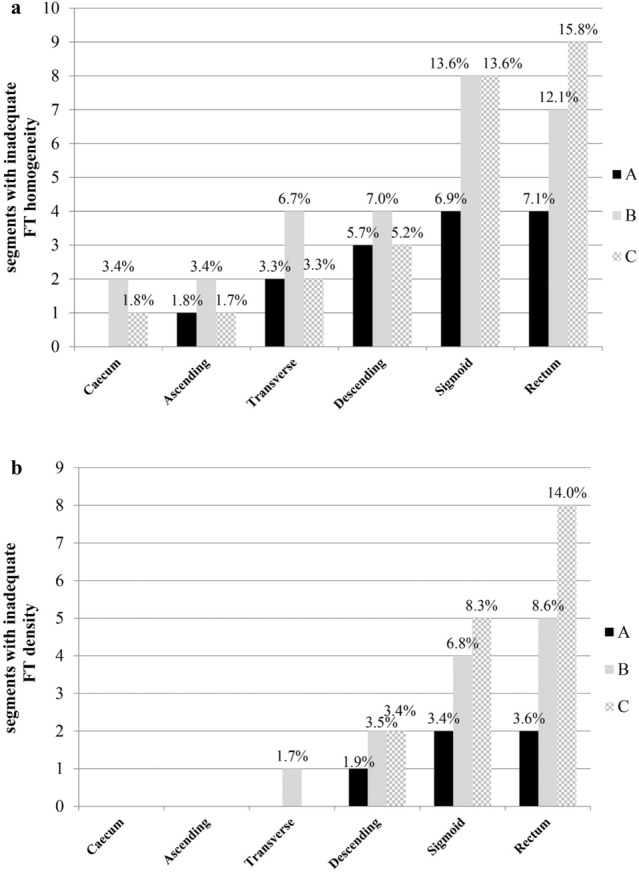
Per-segment qualitative assessment of FT for the three regimens: tagging was considered “inadequate” when a score of 1 for density and/or 1–2 for homogeneity was assigned. The number of inadequate segments for each regimen (columns) is reported on the vertical axis, while the segment type on the abscissa. The value on the top of each column represents the percentage of segments that are inadequate for that type of segment, within the same regimen. All 65 segments with inadequate FT were inadequate for homogeneity (**a**) and 32 of them (49.2%) were inadequate for density also (**b**)

In brief, per-segment analysis was possible in 1042 of the 1080 segments (96.5%). The remaining 38 segments (3.5%) were not evaluable due to the absence of fecal residues in the colon lumen. Figure 4 summarizes quality assessment finding. Overall, the percentage of segments with inadequate FT homogeneity was significantly lower in regimen A with respect to regimen B [4.1% (14/341) vs 7.7% (27/350); *p* = 0.048]; no significant differences were observed between regimen C [6.8% (24/351)] and the other regimens (Fig. 4a). The percentage of segments with insufficient FT density was significantly lower in regimen A than in regimen C [1.5% (5/341) vs 4.3% (15/351); *p* = 0.018]; no significant differences were observed between regimen B [3.4% (12/350)] and the other regimens (Fig. 4b). Inhomogeneous FT was observed in 65 of the 1042 segments (6.2%); among these, 32 also had an inadequate density score, while no segment was considered inadequate based on FT density exclusively.

In a segment-by-segment analysis, the median density scores of the sigmoid colon (*p* = 0.01) and rectum (*p* = 0.02) were significantly different between regimens A and C [median scores of sigmoid colon and rectum in regimen A were 3 (1st and 3rd quartile: 3–3), while in regimen C were 3 (2–3)] (Table 3a). In the segment-by-segment comparison, the median homogeneity scores were significantly different in regimens A and C in the ascending [4 (4–4) and 4 (3–4), respectively; *p* = 0.01], descending [4 (4–4) and 4 (3–4); *p* = 0.01] and sigmoid colon [4 (4–4) and 4 (3–4); *p* = 0.01]. Finally, significant homogeneity differences were also observed between regimens A and B exclusively in the sigmoid colon [4 (4–4) and 4 (3–4); *p* = 0.02] (Table 3b).

**Table 3:** Distribution of qualitative median scores for density (a) and homogeneity (b) across segments and regimens; interquartile ranges in parentheses

a						
Regimen	Caecum	Ascending	Transverse	Descending	Sigmoid	Rectum
A	3 (3–3)	3 (3–3)	3 (3–3)	3 (3–3)	3 (3–3)	3 (3–3)
B	3 (3–3)	3 (3–3)	3 (3–3)	3 (3–3)	3 (3–3)	3 (3–3)
C	3 (3–3)	3 (3–3)	3 (3–3)	3 (3–3)	3 (2–3)	3 (2–3)

b						
Regimen	Caecum	Ascending	Transverse	Descending	Sigmoid	Rectum
A	4 (4–4)	4 (4–4)	4 (4–4)	4 (4–4)	4 (4–4)	4 (3.5–4)
B	4 (3–4)	4 (4–4)	4 (4–4)	4 (3–4)	4 (3–4)	4 (3–4)
C	4 (3–4)	4 (3–4)	4 (3–4)	4 (3–4)	4 (3–4)	4 (3–4)

In the per-patient analysis, CTC was classified non-diagnostic in 22 of the 180 patients (12.2%) due to inadequate homogeneity. Sixteen of the 22 patients (72.7%) were also inadequate for FT density. The number of non-diagnostic CTC scans was not statistically different between the three regimens [6.7% (4/60) in regimen A; 15% (9/60) in regimen B; 15% (9/60) in regimen C; *p* = 0.146].

The interobserver agreement between the two radiologists was excellent for both the FT density [Cohen’s *K* = 0.887 (95%CI 0.857–0.917)] and homogeneity [Cohen’s *K* = 0.852 (95%CI 0.824–0.880)].

### Quantitative assessment

**Fig. 5:**
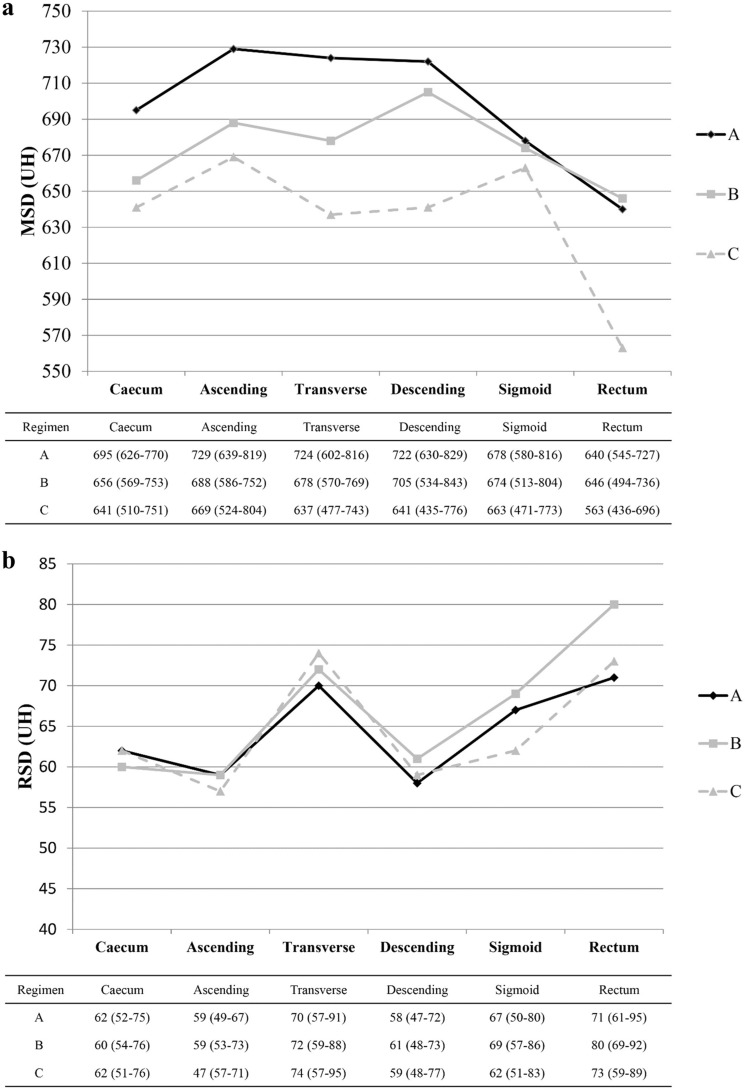
Segment-by-segment comparison between the median scores ​​of MSD (a) and RSD (b) of the three regimens. Each regimen is represented by a different row. The values of MSD (**a**) and RSD (**b**) are reported in the vertical axis and in the respective table, while the segment type on the abscissa. Interquartile ranges are reported in parentheses. MSD Mean Segment Density, RSD Relative Standard Deviation

MSD and RSD were measured in 1001 of the 1080 segments (92.7%; 335 regimen A, 333 regimen B, 333 regimen C). The remaining 79 segments were not evaluated due to the absence of fecal residues (*n* = 38) or tagged fecal residues too small to be evaluated (ROI < 0.5 cm^2^; *n* = 41). Figure 5 shows the results of MSD and RSD analysis. In the segment-by-segment comparison, MSD values were significantly higher in regimen A than in regimen C in all segments (*p* < 0.05), except within the sigmoid colon. A significant difference was also observed between regimens A and B, but only for the ascending colon (*p* = 0.04) (Fig. 5a). There were no statistically significant differences in RSD values between the three regimens across all segments (Fig. 5b).

In the subgroup of patients (10 per group) in which the two sets of ROIs were drawn by the two radiologists, the interobserver variability was not statistically significant for both MSD (*p* = 0.525) and RSD (*p* = 0.420) measures.

### Patient acceptance

**Fig. 6:**
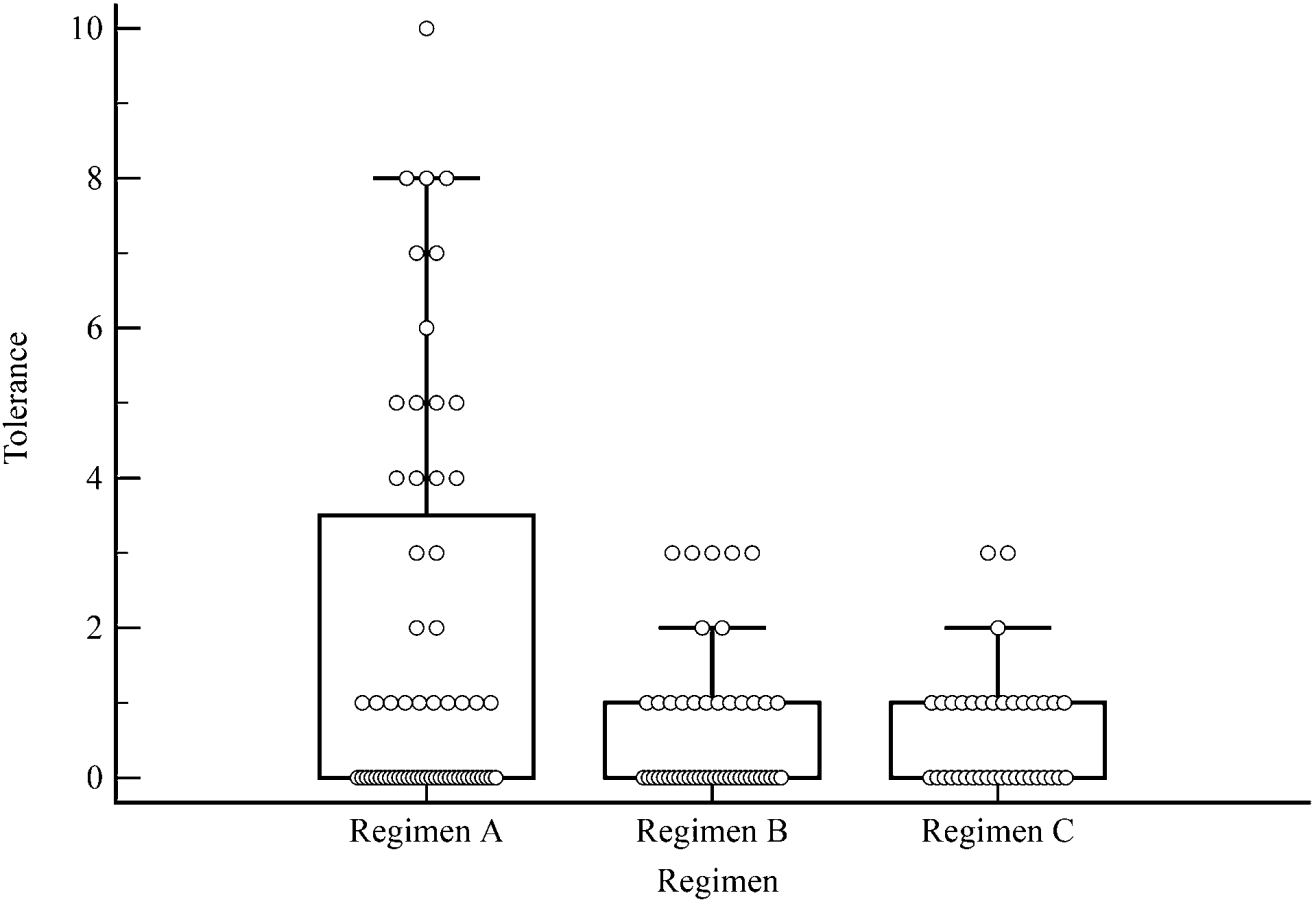
Box plot of tolerance distribution scores for each regimen. The bottom edge of the box represents the first quartile, which in this case overlaps the median score; the top edge of the box represents the third quartile. The lines extending from the boxes (whiskers) indicate the variability outside the upper and lower quartiles, excluding outliers which are defined as those values outside 1.5 times the interquartile range

All three bowel preparations were well tolerated. However, regimen A showed a wider distribution of acceptance values [median scores: 0 (0–3) regimen A; 0 (0–1) regimen B; 0 (0–1) regimen C], without reaching statistical significance (Fig. 6).

### Impact of patient-dependent parameters on CTC quality

Dolichocolon was present in 74 of 180 patients (41%), colonic diverticulosis in 72 (40%), while 32 were diagnosed with functional constipation (18%) and 27 with secondary constipation (15%). Regardless of the regimen administered, a significantly higher number of “inadequate segments” was observed in patients with dolichocolon (5.4% vs 1.6% for FT density and 10.3% vs 3.4% for FT homogeneity, *p* < 0.01) and secondary constipation (9% vs 2.1% for FT density and 11% vs 5.4% for FT homogeneity, *p* < 0.01). The other patient variables (functional constipation and diverticulosis) did not affect the examination quality (Table 4).

**Table 4:** Number of inadequate segments according to patient features

	Dolichocolon		Functional constipation		Causes of secondary constipation		Colonic diverticulosis	
	yes	no	yes	no	yes	no	yes	no
Total segments	429	613	184	858	155	887	421	621
Segments with inadequate FT density, *n* (%)	23 (5.4)	10 (1.6)	10 (5.4)	23 (2.7)	14 (9)	19 (2.1)	4 (0.9)	29 (4.7)
Segments with inadequate FT homogeneity, *n* (%)	44 (10.3)	21 (3.4)	16 (8.7)	49 (5.7)	17 (11)	48 (5.4)	14 (3.3)	51 (8.2)

## Discussion

In this study, we compared CTC examination quality of three different low-volume preparations differing only in the scheduling of the oral PEG solution administration. We found that the administration of a single dose of 120 g PEG solution the day before CTC (regimen A) yielded a lower number of segments with “inadequate FT” compared to the PEG administration in more days (regimens B and C). Indeed, both qualitatively and quantitatively regimen A was found to be the best of the three regimes in terms of FT density and homogeneity. At the best of our knowledge, these results represent a significant finding, since the literature has mainly focused on the timing of fecal tagging regimens [19, 22] or comparing different laxative solutions for colon cleansing [11, 13, 15–17]. Of note, in this study, iodine solution was not a variable since it was always administered orally 2.5 h before CTC.

All three regimens evaluated in this study were affected by a small percentage of poor-quality CTCs, as reported in other low-volume PEG-based regimens [8, 13]. In a previous experience, we found similar results by administering three sachets/day of a Macrogol 3350-based mild laxative starting 2 days before CTC (4.4% of insufficiently marked segments) [13]. Iafrate et al. [8] reported 2.6% of colon segments with insufficient preparation using a 1L PEG solution associated with a stimulant agent (bisacodyl) that is correlated with a higher stool cleansing ability [14]. Both in our experience and in that of the two above-mentioned studies, FT quality decreased in the distal colonic segments. We therefore hypothesized that other factors could affect the quality of the CTC scans, independently of the bowel preparation scheme used. Indeed, we found a significantly higher number of inadequate segments in patients with dolichocolon or secondary constipation (Table 4) [23–25]. Conversely, the number of inadequately tagged segments was not significantly greater in patients with functional constipation and colonic diverticulosis. We therefore hypothesize that in some categories of patients, i.e., those with dolichocolon and secondary constipation, a longer interval between iodine administration and the CTC or an additional laxative could allow a better-quality examination especially of the distal colonic segments.

All regimens were well tolerated probably due to high palatability of the Macrogol solution which is tasteless and soluble in any type of liquid. However, the distribution of acceptance values was slightly wider in regimen A. Indeed, since regimen A is characterized by the administration of a full dose of Macrogol solution in a relatively short span of time, it could cause a greater discomfort to the patients. Indeed, previous literature shows increased patient tolerance as the volume of the administered laxative solution decreases [11]. However, we believe that administering the entire dose of laxative in a single dose could be more easily managed by the patient without significantly affecting his daily routine.

Interobserver variability on both qualitative and quantitative parameters showed high agreement between the two readers with no statistically significant differences between the two ROIs sets, showing that the methods were reliable and reproducible.

This study has some limitations. First, the sample size of the study was not sufficiently powered to compare per-patient examination quality across preparations. Second, qualitative analysis was affected by subjectivity. However, we amended to the above-mentioned by performing a consensus evaluation in case of discordant results. Moreover, the agreement between the two radiologists was very good for both FT homogeneity and density. Third, MSD and RSD were measured by one of the two radiologists in each colonic segment by means of a manually drawn ROI over the area with the greatest quantity of tagged fluids. This methodological approach is not entirely reproducible, as it is influenced by radiologist’s preferences, and is a time-consuming process. However, measurements were extended to multiple colonic segments, a process which in our opinion mitigates variability allowing a more accurate and complete analyses. Furthermore, we demonstrated that interobserver variability between two readers was not statistically significant in a subgroup of patients (10 per group). Finally, ROIs smaller than 0.5 cm^2^ were excluded, as they were considered too small to be reliable. In the future, automatic segmentation of tagged fluid will allow more reproducible and less time-consuming measurements.

In conclusion, in this randomized study, we have shown that the best quality CTC examination is obtained by administering 120 g of a PEG solution the day before the examination, as with this preparation regimen, the number of segments with inadequate FT is lower with respect to the fractioned schemes. Moreover, the single dose scheme should affect less patient’s daily routine. In clinical practice, splitting the dose of a low-volume PEG solution could lead to a greater risk of non-adequately tagged segments, thus making it not recommended to let the patient choose which type of regimen to take according to his preferences.

We have identified dolichocolon and secondary constipation, not diverticular disease, and functional constipation, as conditions for increased risk of insufficiently tagged colonic segments. Taking into account patients clinical characteristics could pave the way to more personalized preparation regimens.

## Abbreviations

CTC: Computed tomographic colonography
PEG: Polyethylene glycol
FT: Fecal tagging
MSD: Mean segment density
RSD: Relative standard deviation
ROI: Region of interest
